# Riding the R Train into the Cell

**DOI:** 10.1371/journal.ppat.1005036

**Published:** 2015-08-20

**Authors:** Daniel DiMaio, Christopher G. Burd, Kylia Goodner

**Affiliations:** 1 Department of Genetics, Yale School of Medicine, New Haven, Connecticut, United States of America; 2 Department of Molecular Biophysics and Biochemistry, Yale University, New Haven, Connecticut, United States of America; 3 Department of Therapeutic Radiology, Yale School of Medicine, New Haven, Connecticut, United States of America; 4 Yale Cancer Center, New Haven, Connecticut, United States of America; 5 Department of Cell Biology, Yale School of Medicine, New Haven, Connecticut, United States of America; University of Michigan Medical School, UNITED STATES

A wise traveler arriving in an unfamiliar city would be ill-advised to blaze a new route from the outskirts, but rather should rely on existing, well-lit transit systems to journey to the city center while avoiding the brigands lurking in the shadows. So too, a wise virus entering a cell will exploit the well-established trafficking routes that maintain cellular life.

Incoming virus particles face enormous challenges to initiate infection. After the virion binds and enters the cell, the capsid proteins must transport the viral genome to the site where it is released, expressed, and replicated. This is often a difficult and hazardous journey with membranes to traverse and cellular defenses to avoid. A non-enveloped DNA virus must navigate from the cell surface into the nucleus, where viral gene expression and genome replication occur, while crossing cell membranes, undergoing disassembly, and avoiding cellular innate immune defenses en route [1].

Among the cellular pathways that transport material from the periphery deep into the cell, the vesicular retrograde transport system is a likely candidate for conveying incoming DNA viruses. The Golgi apparatus is a major sorting hub of the cell where newly synthesized proteins are modified and distributed to the cell surface or other locations in the cell, such as the endosome. The transport of proteins from the Golgi apparatus often depends on carrier proteins that ferry cellular cargos to their destinations, a process that depletes the carriers from the Golgi. These carriers are retrieved back to the Golgi in a retrograde fashion for reuse after they have delivered their cargo [2]. Retromer, a conserved cytoplasmic protein complex, plays a central role in retrograde endosome-to-Golgi transport (as well as in endosome-to-plasma membrane transport) [2–4]. Retromer is a complex of three proteins (VPS29, VPS35, and VPS26) that recognizes sorting signals in the cytoplasmic domains of transmembrane proteins at the endosomal membrane. Retromer also associates with sorting nexins—peripheral membrane proteins that bind the endosomal membrane and help shape it into transport vesicles containing the cargo—which bud off of the endosomal membrane. These transport vesicles then travel in a microtubule-dependent fashion to their target organelle, where they fuse with the organelle membrane to deliver the cargo. Thus, cells have an elaborate vesicular system to transfer proteins from the endosome to the Golgi. Certain plant and bacterial toxins utilize this retrograde pathway to enter cells [1,5], so it seems logical that some viruses would also exploit this pathway to make the same journey.

The first evidence implicating retromer in virus entry came from studies of human papillomavirus type 16 (HPV16), a non-enveloped DNA virus. HPV are responsible for approximately 5% of human cancer, but little is known about their trafficking during cell entry. Because the identification of cellular proteins involved in HPV entry would elucidate the trafficking pathway involved in this process and identify possible targets for novel anti-viral approaches, we conducted a genome-wide siRNA screen for cellular factors required for the entry of HPV16 pseudovirus into cultured human epithelial cells [6]. This screen and the follow-up validation experiments identified numerous cellular proteins as being required for HPV16 entry, including several components of the retrograde transport pathway and all three retromer subunits. One explanation for these findings is that HPV16 entry requires one or more cellular proteins that must undergo retromer-mediated retrograde transport. Indeed, retrograde transport systems have been implicated in infection by other viruses. The coatomer complex COPI, which plays a role in trafficking between the Golgi and the endoplasmic reticulum (ER), has been identified in RNA interference screens for cellular factors required for infection by several RNA viruses. However, COPI does not necessarily directly mediate intracellular trafficking of the incoming virus particle but rather may play an indirect role in virus infection. For example, it has been proposed that COPI components are required to establish membrane-associated replication compartments for poliovirus and hepatitis C virus [7,8].

**Fig 1:**
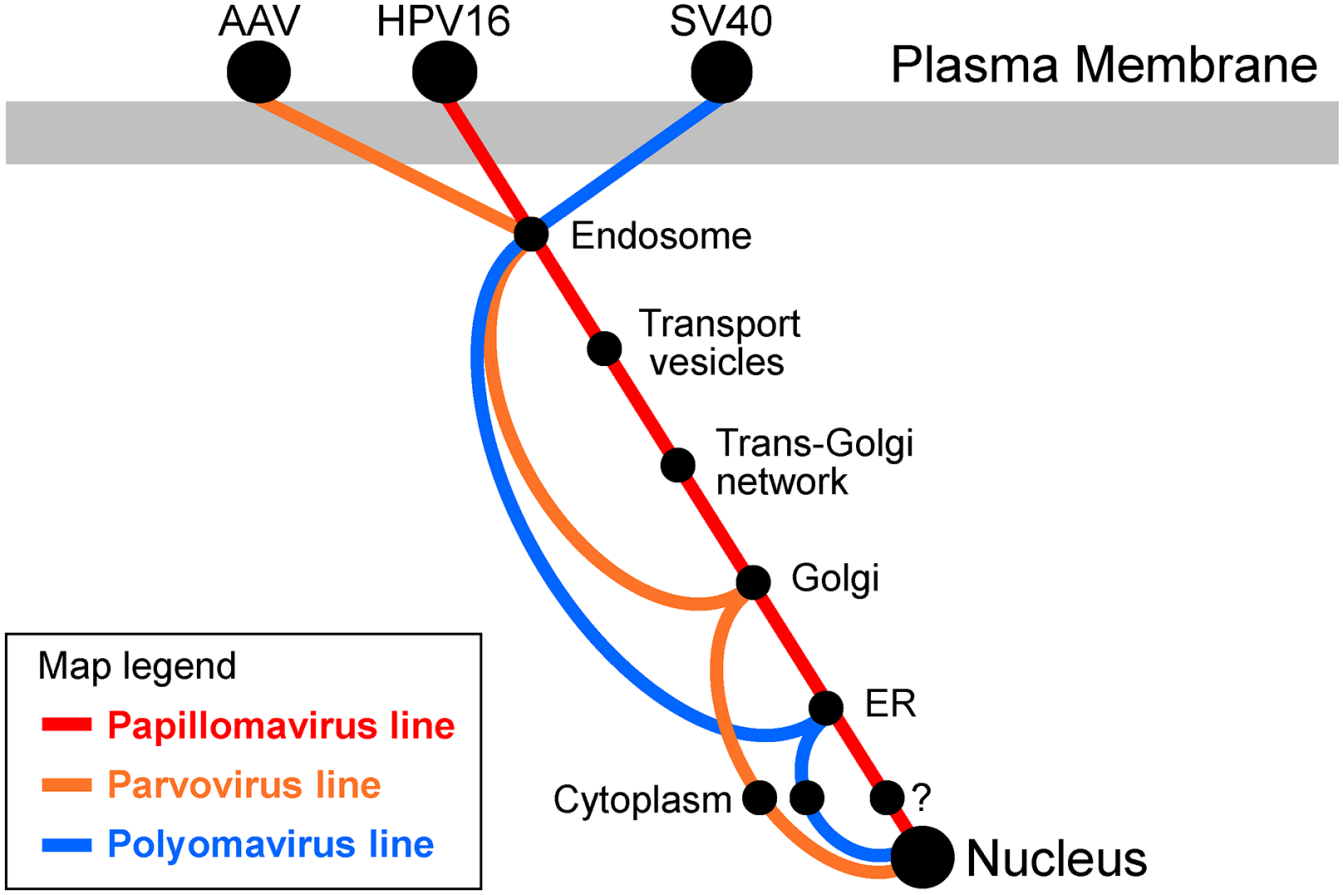
Transit pathways used by small DNA viruses. The map shows our current understanding of the intracellular trafficking pathways used during entry by three families of small DNA viruses: human papillomaviruses (red), parvoviruses (orange), and polyomaviruses (blue). A prototype member of each virus family is shown at the top, where it binds to a distinct receptor at the cell surface. Receptors and vesicular sites implicated in virus entry are represented by the black spots. Parvoviruses and polyomaviruses are thought to exit the vesicular pathway into the cytoplasm prior to nuclear entry. HPV engages the retromer to exit the endosome, but it is unclear if there is a distinct cytoplasmic phase.

Alternatively, incoming HPV16 itself, or infectious components thereof, may undergo retrograde transport. At first glance, this possibility seemed unlikely because HPV lacks transmembrane proteins, which were the only known retromer cargos [3,4]. In addition, the prevailing view in the HPV field was that incoming virus was transported out of the endosome directly into the cytoplasm [9]. However, several recent pieces of evidence suggest that HPV16 is transported in a vesicular fashion by the retrograde pathway during entry. Localization studies revealed viral capsid proteins and viral DNA in membrane-bound organelles prior to entry of viral DNA and the minor capsid protein L2 into the nucleus [6,10]. At early times of infection, viral components are present in the early endosome, and, at later times, in the trans-Golgi network, the Golgi apparatus, and the ER (Fig 1) [11,12]. Virus at these sites appears to be undergoing infectious entry because genetic knockdown of retrograde transport factors (such as Rab proteins) and pharmacologic inhibition of retrograde transport block the delivery of viral components to the distal sites in the pathway and prevent viral gene expression [6,10,13]. Similarly, knockdown or inhibition of the cellular protease γ-secretase blocks infection after the L2 protein and the viral DNA exit the endosome (but prior to their arrival in the Golgi), and some mutations in the L2 protein trap the viral genome in the Golgi and abort infection [12,14]. Finally, TRAPPC8 and SNX17, cellular proteins involved in vesicular trafficking, form stable complexes with HPV L2 and are required for proper intracellular trafficking of HPV16 during entry [15,16].

Most strikingly, knockdown of retromer expression or some mutations in the C-terminal segment of the HPV16 L2 protein causes the accumulation of the L2 protein in the endosome and prevents its arrival in the Golgi [6,17]. The L2 sequences required for endosome exit are highly conserved in different HPV types and resemble sorting motifs in cellular proteins that bind retromer. Indeed, the entry defect of these L2 mutants could be rescued by replacing the viral element with a known retromer sorting signal from a cellular protein [17,18]. Furthermore, the L2 element was able to support the transport of a marker protein from the endosome to the Golgi in a retromer-dependent fashion. Finally, in vitro binding experiments demonstrated that retromer binds directly to the motifs in L2 that are required for endosome exit [17]. Taken together, these findings establish that HPV16 L2 (and presumably associated viral DNA) is a novel type of retromer cargo, which is transported by the retrograde pathway into the Golgi. Although retrograde trafficking of other HPV types has not been studied in detail, all sequenced HPV types contain a retromer binding motif, and recent evidence suggests that multiple high-risk HPV types utilize similar entry pathways [17,19]. It is not known how incoming HPV16 in the endosome lumen engages retromer in the cytoplasm because L2 is not a classic transmembrane protein, but it is possible that the C-terminus of L2, containing retromer binding sites, protrudes through the endosomal membrane into the cytoplasm, where it is bound by retromer. Consistent with this model, this segment of the L2 protein has pH-dependent membrane-destabilizing activity and can target a reporter protein to cell membranes [20].

Although the studies cited above were the first to forge a link between retromer and virus entry, retromer has been implicated in other aspects of virus infection. For the lentivirus HIV-1, intracellular trafficking of newly synthesized envelope glycoprotein (Env) regulates virus assembly during the late stage of infection. Groppelli showed that the cytoplasmic tail of the gp41 Env subunit binds retromer, an interaction that is required for normal Env trafficking [21]. Inhibition of retromer function resulted in increased cell surface expression of Env, increased incorporation of Env into nascent virions, and enhanced infectivity. In addition, the Herpesvirus Saimiri (HVS) tyrosine kinase-interacting oncoprotein Tip binds to retromer and inhibits its retrograde transport activity [22]. This ultimately results in down-regulation of cell surface CD4 expression and enhanced in vitro immortalization activity of HVS in primary human T cells.

HPV are not the only DNA viruses that engage the retrograde transport system during entry. Polyomaviruses such as SV40 and JC virus have long been known to transit to the ER, where important capsid disassembly steps take place [23–25]. Polyomavirus trafficking to the ER is inhibited by pharmacologic inhibitors of retrograde transport [26] and by inhibition of COPI function [27,28]. However, polyomaviruses have not been detected in the Golgi apparatus and polyomavirus entry does not require retromer, so the route these viruses take to the ER is not known (Fig 1) [12,29]. The parvovirus Adeno-associated virus (AAV) also utilizes the retrograde pathway during intracellular trafficking. After internalization, AAV travels in maturing endosomal compartments to the Golgi apparatus [30], prior to exit into the cytoplasm (Fig 1). Golgi trafficking and infectivity of AAV in epithelial cells are inhibited by chemical inhibitors of retrograde transport or by knockdown of the tSNARE syntaxin 5 (STX5), but do not require retromer or retrograde transport factors Rab7, Rab9, Rab11, STX6, or STX16 [31]. These results suggest that AAV utilizes a novel retrograde transport pathway to enter epithelial cells. In neurons, retrograde transport of AAV9 appears to require Rab7, so the mechanism of AAV trafficking may show important cell type differences [32,33].

A vesicular retrograde entry pathway provides several benefits to incoming virus particles. First, it might be the most direct or efficient route to a crucial intracellular location while targeting the virion away from dead-end sites, such as the lysosome, where degradation would occur. Second, it might allow the incoming capsid to reach a vesicular location where some other essential entry process or factor resides, such as enzymes that catalyze crucial capsid rearrangement or proteolytic cleavage events, or factors that mediate membrane penetration or nuclear import. Finally, a vesicular route will shield the virus and viral DNA from cytoplasmic innate immune sensors that might otherwise inhibit infection. Eventually, the viral genome must leave the retrograde pathway and enter the nucleus. This step presumably involves one or more membrane penetration or membrane fusion events and may include the passage of viral components through the cytoplasm, but consideration of this important topic is outside the scope of this brief review.

The results summarized above demonstrate that viruses can traffic during entry via non-canonical retrograde transport pathways or via known retrograde pathways in unusual ways. Thus, further analysis of virus entry will elucidate novel features of cellular trafficking pathways and processes. Because these processes may well be utilized by the cell to sort cellular proteins, as well as to mediate virus trafficking, studies of virus entry are likely to provide unexpected new insights into fundamental cell biology.
